# Assessment of the E-value in the presence of bias amplification: a simulation study

**DOI:** 10.1186/s12874-024-02196-4

**Published:** 2024-03-28

**Authors:** Eric Barrette, Lucas Higuera, Kael Wherry

**Affiliations:** Medtronic, Plc, Minneapolis, MN USA

**Keywords:** Propensity score matching, E-value, Residual confounding, Simulation

## Abstract

**Background:**

The E-value, a measure that has received recent attention in the comparative effectiveness literature, reports the minimum strength of association between an unmeasured confounder and the treatment and outcome that would explain away the estimated treatment effect. This study contributes to the literature on the applications and interpretations of E-values by examining how the E-value is impacted by data with varying levels of association of unobserved covariates with the treatment and outcome measure when covariate adjustment is applied. We calculate the E-value after using regression and propensity score methods (PSMs) to adjust for differences in observed covariates. Propensity score methods are a common observational research method used to balance observed covariates between treatment groups. In practice, researchers may assume propensity score methods that balance treatment groups across observed characteristics will extend to balance of unobserved characteristics. However, that assumption is not testable and has been shown to not hold in realistic data settings. We assess the E-value when covariate adjustment affects the imbalance in unobserved covariates.

**Methods:**

Our study uses Monte Carlo simulations to evaluate the impact of unobserved confounders on the treatment effect estimates and to evaluate the performance of the E-Value sensitivity test with the application of regression and propensity score methods under varying levels of unobserved confounding. Specifically, we compare observed and unobserved confounder balance, odds ratios of treatment vs. control, and E-Value sensitivity test statistics from generalized linear model (GLM) regression models, inverse-probability weighted models, and propensity score matching models, over correlations of increasing strength between observed and unobserved confounders.

**Results:**

We confirm previous findings that propensity score methods – matching or weighting – may increase the imbalance in unobserved confounders. The magnitude of the effect depends on the strength of correlation between the confounder, treatment, and outcomes. We find that E-values calculated after applying propensity score methods tend to be larger when unobserved confounders result in more biased treatment effect estimates.

**Conclusions:**

The E-Value may misrepresent the size of the unobserved effect needed to change the magnitude of the association between treatment and outcome when propensity score methods are used. Thus, caution is warranted when interpreting the E-Value in the context of propensity score methods.

## Background

Observational data or “real-world data” (RWD) are data including administrative healthcare claims, electronic health records (EHR), non-randomized registries, and patient data collected via mobile applications or wearable devices that offer many advantages for research [1]. However, observational data also present challenges for researchers. Most notably, treatments or interventions are rarely randomly assigned outside of clinical trial settings, and patient populations often include everyone treated in the usual course of care without the specific inclusion and exclusion criteria of a clinical trial. Many times, the observable covariates between people who select the intervention and people who do not select the intervention are unbalanced.

Common analytic methods for achieving unbiased treatment effect estimates, such as propensity score matching, inverse probability of treatment weighting (using propensity scores), and regression-based approaches can adjust for differences in observed covariates. Propensity score methods (PSMs) such as matching and weighting have the benefit of producing balance in observable covariates between treated and untreated groups, analogous to a randomized study [2]. In practice, researchers may assume PSM-induced balance between treatment groups across observed characteristics will extend to balance of unobserved characteristics. However, this assumption is not testable and has been shown to not always be true. [3]. Achieving unbiased estimates with PSMs or regression-based methods is predicated on the assumption of “strong ignorability” or ‘unconfoundedness”, that is, that given observed covariates, treatment assignment is independent of the potential outcomes [4]. Unfortunately, with any of these methods it is impossible to directly test if unobserved covariates are related to the treatment and the outcome, not to mention balanced. Moreover, prior research has shown that using PSMs to balance observed covariates can result in more biased treatment effect estimates, compared to non-PSMs, by increasing the imbalance in unobserved covariates.

Numerous approaches, i.e., “sensitivity analyses”, have been proposed to assess the potential impact of unmeasured confounders [5–8]. One technique growing in popularity is the E-value, defined as “the minimum strength of association, on the risk ratio scale, that an unmeasured confounder would need to have with both the treatment and outcome, conditional on the measured covariates, to fully explain away a specific treatment” [9]. Unlike many sensitivity tests, the E-value does not require assumptions about the number of unmeasured confounders or their functional form. The E-value is also appealing due to the direct calculation from a risk ratio or an approximation of a risk ratio from other common treatment effect estimates (e.g., odds ratios or hazard ratios). However, if the treatment effect estimate is biased, the effect on the E-value and its subsequent interpretation is not obvious. Our study contributes to the growing literature on the applications and interpretations of E-values, and by extension the sensitivity analysis literature. Specifically, this study sought to answer the question of how the performance of the E-value is impacted in simulated data with varying levels of association between unobserved covariates and treatment and outcome.

### Conceptual background

Our simulation study is tangentially related to the bias amplification literature. That literature considers the effect of conditioning on variables that are associated with treatment but not the outcome (except through treatment) – also known as instrumental variables – or variables that are much more strongly associated with treatment than the outcome – sometimes referred to as “near instruments”. Theoretical results and simulation studies have shown that controlling for an instrumental variable causes bias in treatment effect estimates [10, 11]. Potential bias amplification is an important consideration when designing an observational analysis because it has been shown through simulation studies to occur in a variety of realistic models [3, 12–15]. Our study diverges from the bias amplification literature by considering data with an unobserved covariate associated with treatment only. If this type of covariate was observed, it would be an instrumental variable but in our simulated data, it is not available to the researcher.

Our choice of this data structure is two-fold. First, previous research has used this structure in simulations and found that imbalance in the portion of the variation of the unobserved covariates that affect treatment choice that is independent of the observed covariates is necessary for propensity score-based methods to achieve balance in observed covariates. However, achieving balance also leads to greater imbalance in unobserved covariates and subsequently results in more biased treatment effect estimates [16]. Second, we contend, as did the researchers who used it previously, that this data structure is not uncommon. Consider a hypothetical population of patients with diabetes. The treatment is use of an insulin pump versus multiple daily injections of insulin. The outcome of interest could be a discrete measure of whether blood glucose time in target range was achieved or not. Characteristics associated with both treatment and outcome like age would be observed. Other demographic or socioeconomic characteristics may also be associated with both treatment and outcome but not observed. Finally, there is some other unobserved factor related only to the probability of using an insulin pump, such as physicians’ preference.

Our simulation seeks to assess how the E-value magnitude varies relative to a treatment effect estimate that has varying degrees of bias. It has been shown that the E-value has a nearly linearly monotonic relationship to a treatment effect estimate. Thus, for a given treatment effect estimate value the E-value is always the same, no matter the research setting, data, or analysis method used [17]. Moreover, the derivation of the E-value assumes that unmeasured covariates are equally related to the treatment and outcome [9]. This is an assumption that has been contested by other researchers as being unlikely in many settings [18]. Using simulated, but realistic, data we are able to vary the strengths of associations in unobserved covariates between treatment and outcome. To provide practical results for practitioners we include commonly used treatment effect estimation methods: regression and PSMs to control for observed covariates.

## Methods

We test the relationship between the E-value (and potential conclusions drawn from the E-value) and propensity score methods under varying scenarios of unobserved confounding. Using Monte Carlo methods we simulate a simple dataset including observed and unobserved covariates with varying levels of correlation between treatment and outcome based on the model in Brooks and Ohsfeldt [3]. This published model shows the tradeoffs between balance and bias in PSMs, and offers an appropriate framework to test how E-values handle unobserved confounders in a realistic approximation of observational research. First, we report the estimated odds ratio of the effect of treatment on the outcome relative to a control across various correlation scenarios and propensity-score methods (inverse-probability weighted models and propensity score matching, based on the same propensity score). Next, we compare observed and unobserved covariate balance across the simulated scenarios. Finally, to assess potential conclusions about study robustness to unobserved covariates, we evaluate the calculated E-values across correlation and PSM scenarios.

### Model

As in Brooks and Ohsfeldt [3], a patient’s net utility gain from treatment ($Tx$) depends on the value of being cured ($V$), the relative cost of treatment ($S$), an observed confounder (${X}_{m}$), and a set of unobserved confounders (${X}_{u1},{X}_{u3},{X}_{u4}$).

1 $$Tx=V{\beta }_{T}-S+\alpha {X}_{m}+\alpha {X}_{u1}+\alpha {X}_{u3}+\alpha {X}_{u4}$$

Parameter $$\alpha$$ weights how confounders affect treatment decision, and $${\beta }_{T}$$ denotes how treatment affects the likelihood of being cured; this is our parameter of interest. The distributions and correlations of $$Xs$$ are described below. A patient is treated ($$T=1)$$ if $$Tx>0$$, and it is not ($$T=0)$$ otherwise.

The probability of a patient being cured depends on treatment $T$, the observed confounder ${X}_{m}$, and a set of unobserved confounders ${X}_{u1},{X}_{u2}$.

2 $${\text{Pr}}\left(C\right)=\frac{{\text{exp}}({\beta }_{T}T+{\beta }_{m}{X}_{m}+{\beta }_{u1}{X}_{u1}+{\beta }_{u2}{X}_{u2})}{1+{\text{exp}}({\beta }_{T}T+{\beta }_{m}{X}_{m}+{\beta }_{u1}{X}_{u1}+{\beta }_{u2}{X}_{u2})}$$

A patient is cured ($C=1$) based on a Bernoulli distribution with probability ${\text{Pr}}(C)$.

### Data and simulations

All data are simulated in this study. We used the same distributions in Brooks and Ohsfeldt to make our results comparable to theirs. There is one observed confounder ${X}_{m}$ drawn from a uniform [0,1] distribution, while unobserved confounders ${X}_{u1},{X}_{u3},{X}_{u4}$ are linear combinations of ${X}_{m}$ and $\mu$, a random variable distributed uniform [0,1], weighted by a correlation $\rho \ge 0$.

3 $${X}_{ui}=\rho {X}_{m}+\left(1-\rho \right)\mu$$

The remaining unobserved confounder ${X}_{u2}$ is a linear combination between unobserved confounder ${X}_{u3}$ and $\mu$.

4 $${X}_{u2}=0.5{X}_{u3}+0.5\mu$$

Note that the unobserved confounder ${X}_{u1}$ affects both the treatment decision and the probability of cure, and it is the main source of bias in the model. However, the correlation between ${X}_{u2}$ and ${X}_{u3}$ introduces an indirect path between treatment decision and the probability of cure. We use these confounders to generate $T$ and $C$ according to Eqs. (1) and (2), respectively. We generate 1,000 random datasets with 10,000 observations each for values of $\rho =\left\{\mathrm{0,0.1,0.2},\dots ,0.9\right\}$.

### Estimation

For each dataset we estimate ${\beta }_{T}$ and its associated risk ratio from a series of generalized linear regressions with $C$ as outcome, binomial family and logit link, and 1) no additional confounders, 2) ${X}_{m}$, or observed confounders only, and 3) ${X}_{m},{X}_{u1},{X}_{u2}$, or observed and unobserved relevant confounders. For the propensity score methods, we estimate 1) an inverse probability weighted generalized linear regression, weighted by the inverse of a probability of treatment predicted from a probit model with ${X}_{m}$ as control, and 2) a 1:1 greedy propensity score matched model with a caliper of 0.001; as a sensitivity analysis, we estimate 1:1 propensity score matching models with less restrictive calipers of 0.1 and 0.01. These methods reflect current practices in observational research, where an association –with and without a causal interpretation- of a treatment with an outcome is estimated using only observed confounders. We also calculate the Standardized Mean Difference (SMD) for ${X}_{m},{X}_{u1},{X}_{u2}$ without any adjustments and with the propensity score methods. Lastly, we use the estimated risk ratios (RR) to calculate the E-Value as in VanderWeele and Ding [9]:

$$E=RR+{\left(RR\times \left(1-RR\right)\right)}^\frac{1}{2}$$

## Results

**Fig. 1:**
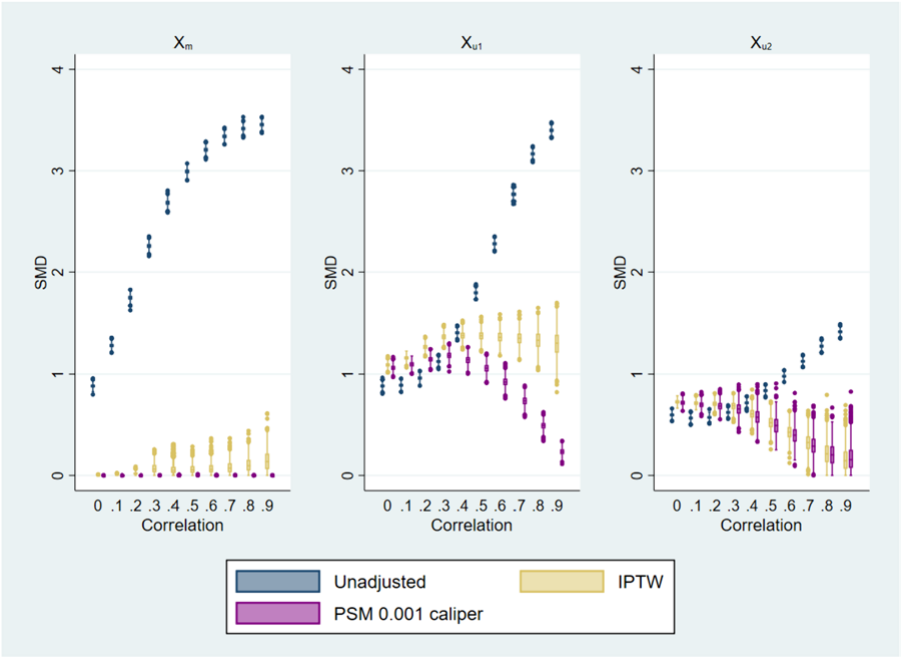
Balance of observable X_m and unobservables X_u1, X_u2 before and after adjustment. Note: Median, interquantile range box, and outliers of the standardized mean differences after 1,000 simulations. *SMD* standardized mean difference. *IPTW* Inverse probability treatment weighting. *PSM* Propensity score matching

Figure 1 shows the SMD of observables (${X}_{m}$) and unobservables (${X}_{u1}$ and ${X}_{u2}$) of the results of the Monte Carlo simulations by method (unadjusted, inverse probability of treatment weighting (IPTW), and PSM) and correlation $\rho$. For the observed confounder ${X}_{m}$, at all correlation levels both IPTW and PSM successfully improve balance with respect to the unadjusted results; however, at higher correlations the balance of IPTW worsens.

Compared to the unadjusted results, the SMD of ${X}_{u1}$ is higher with IPTW and PSM when the correlation is low. For higher correlations, the SMDs from IPTW and PSM are lower than the unadjusted, but only the SMD with PSM decreases as correlation increases. The SMDs of ${X}_{u2}$ follow a similar pattern, where the SMDs from IPTW and PSM are higher with respect to the unadjusted results at lower correlations, but these SMDs decrease when correlation increases. Except for the observed confounder ${X}_{m}$, neither IPTW nor PSM achieve SMDs to the informal level of 0.1 to consider the imbalance corrected.

**Fig. 2:**
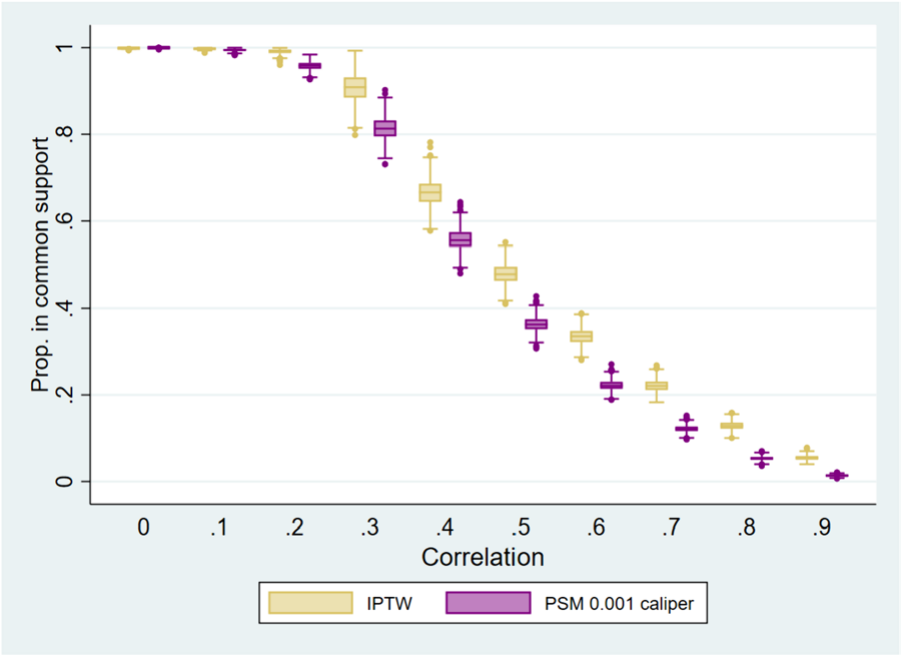
Proportion of observations in common support. Note: Median interquantile range box, and outliers of the proportion of observations in common support after 1,000 simulations results. *OR* Odds ratio. *IPTW* Inverse probability treatment weighting. *PSM* Propensity score matching

Figure 2 shows the proportion of observations in common support from IPTW and PSM, defined as observations with an overlapping estimated probability of treatment. At lower correlations, few observations are outside the common support, but at higher correlations observations in common support reduce rapidly. Median observations in the common support are lower in PSM than in IPTW at all correlations greater than 0.

The results of the regression specifications applying IPTW or PSM are reported in Table 1.

**Table 1:** Model results, by correlation and estimation method

$\rho$	All confounders		No confounders		Observed confounder		IPTW		PSM caliper 0.001	
	$\widehat{{\beta }_{T}}$	RR	$\widehat{{\beta }_{T}}$	RR	$\widehat{{\beta }_{T}}$	RR	$\widehat{{\beta }_{T}}$	RR	$\widehat{{\beta }_{T}}$	RR
0	0.197 (0.057)	1.069 (0.021)	0.310 (0.043)	1.110 (0.016)	0.262 (0.048)	1.092 (0.018)	0.263 (0.049)	1.093 (0.018)	0.267 (0.064)	1.095 (0.024)
0.1	0.202 (0.057)	1.071 (0.020)	0.327 (0.041)	1.117 (0.016)	0.258 (0.049)	1.091 (0.018)	0.261 (0.053)	1.092 (0.019)	0.263 (0.072)	1.094 (0.027)
0.2	0.206 (0.064)	1.072 (0.023)	0.343 (0.042)	1.123 (0.016)	0.258 (0.057)	1.091 (0.021)	0.260 (0.071)	1.091 (0.027)	0.262 (0.090)	1.092 (0.033)
0.3	0.202 (0.067)	1.070 (0.024)	0.358 (0.041)	1.129 (0.016)	0.247 (0.062)	1.086 (0.023)	0.271 (0.108)	1.097 (0.040)	0.264 (0.142)	1.093 (0.053)
0.4	0.198 (0.073)	1.069 (0.027)	0.370 (0.042)	1.133 (0.016)	0.233 (0.069)	1.082 (0.025)	0.287 (0.110)	1.102 (0.041)	0.239 (0.154)	1.084 (0.057)
0.5	0.201 (0.074)	1.070 (0.027)	0.383 (0.043)	1.138 (0.017)	0.224 (0.073)	1.078 (0.027)	0.314 (0.109)	1.112 (0.041)	0.242 (0.173)	1.085 (0.064)
0.6	0.201 (0.080)	1.070 (0.029)	0.393 (0.043)	1.142 (0.017)	0.217 (0.079)	1.076 (0.029)	0.342 (0.108)	1.122 (0.041)	0.246 (0.195)	1.086 (0.072)
0.7	0.200 (0.085)	1.070 (0.031)	0.401 (0.043)	1.145 (0.017)	0.206 (0.085)	1.072 (0.031)	0.366 (0.097)	1.131 (0.038)	0.236 (0.233)	1.081 (0.087)
0.8	0.200 (0.086)	1.070 (0.031)	0.414 (0.043)	1.150 (0.017)	0.203 (0.086)	1.070 (0.031)	0.384 (0.089)	1.138 (0.035)	0.217 (0.309)	1.074 (0.119)
0.9	0.204 (0.084)	1.071 (0.030)	0.426 (0.043)	1.155 (0.017)	0.205 (0.084)	1.071 (0.030)	0.411 (0.070)	1.148 (0.027)	0.235 (0.491)	1.079 (0.188)

Median and standard deviation of coefficients and Odds ratios of 1,000 simulations results. RR: Risk ratio. IPTW: Inverse probability treatment weighting. PSM: Propensity score matching

**Fig. 3:**
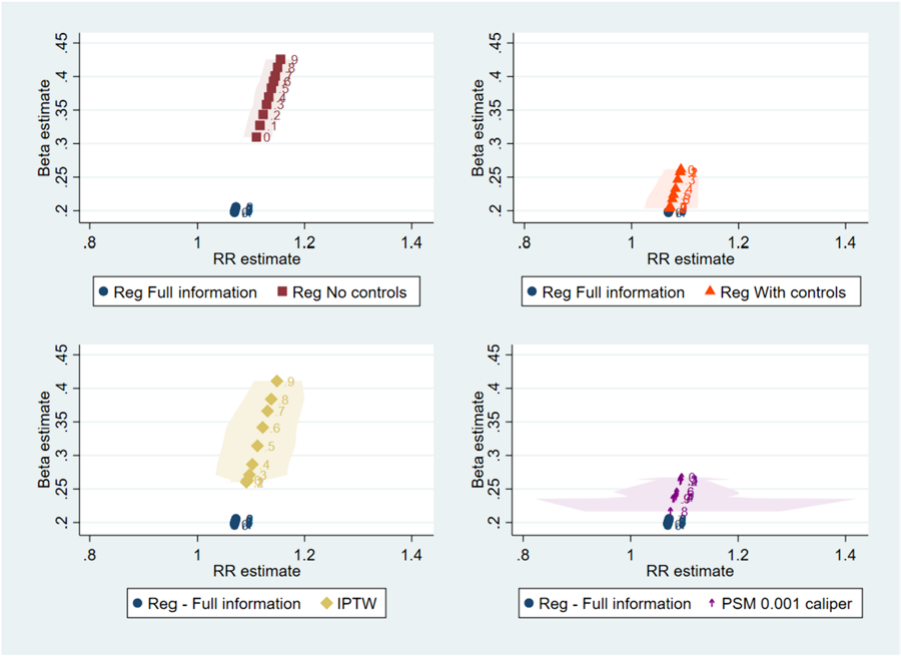
Coefficient estimates and Risk Ratios. Note: Median (marker) and x-axis percentiles 5 and 95 (shaded area) of 1,000 simulations results. *RR* Risk ratio. *IPTW* Inverse probability treatment weighting. *PSM* Propensity score matching

In Fig. 3, we plot the estimated coefficients and associated risk ratios of each estimate against the full information regression. The full information regressions correctly estimate ${\beta }_{T}$ at 0.2, with an associated risk ratio of 1.07. The results from the regression without confounders (upper left side of Fig. 3) show an upwardly biased $\widehat{{\beta }_{T}}$, and this bias increases with a higher correlation. IPTW results show a similar pattern, with an overall biased $\widehat{{\beta }_{T}}$, and a higher bias with a higher correlation. But there is more variability in the $\widehat{{\beta }_{T}}$ estimate with IPTW compared to the regression specification without confounders. Conversely, the regression with ${X}_{m}$ as control (upper right side of Fig. 3) shows a biased $\widehat{{\beta }_{T}}$ at lower correlation levels, but this bias decreases as the correlation increases. Results from PSM specifications also show a decrease in bias at higher levels of correlation, and a substantially higher variability at higher correlations.

**Fig. 4:**
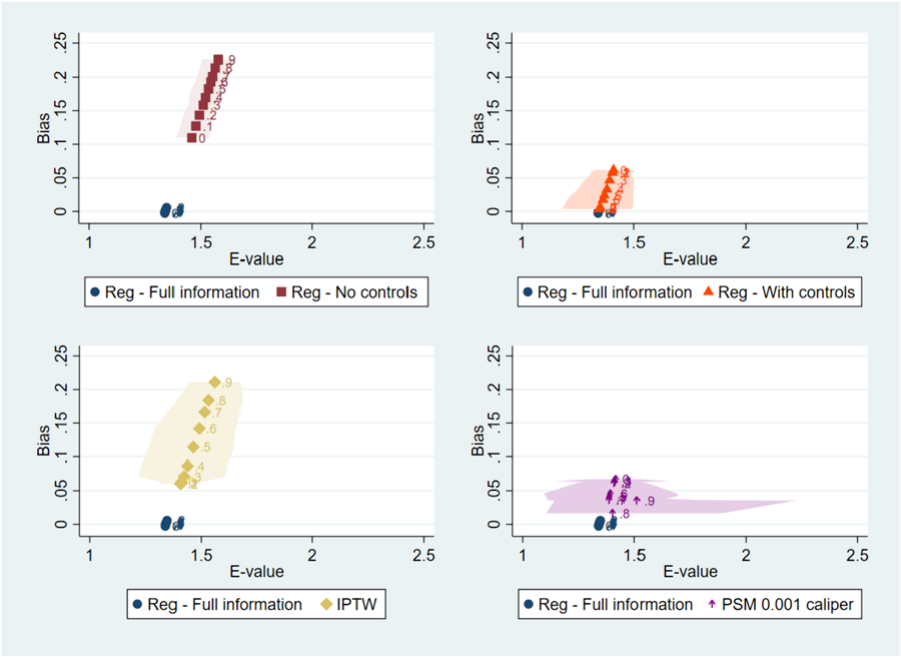
Bias and E-Value. Note: Median (marker) and x-axis percentiles 5 and 95 of 1,000 simulations results. Bias defined as β_T-β ^_T. *IPTW* Inverse probability treatment weighting. *PSM* Propensity score matching

In Fig. 4 we plot median E-values and bias in the estimation of ${\beta }_{T}$ (defined as the difference between ${\beta }_{T}$ and ${\widehat{\beta }}_{T}$) by level of correlation for each estimation method, compared to the full information regression. There is a positive association between E-values and bias: larger E-values are paired to larger differences between the estimated risk ratio and the true risk ratio. As in the previous results, higher correlations between the observable and unobservable confounders increase the E-values in the regressions without controls and in IPTW specifications and decrease the E-values in the regressions with controls and in PSM. The variability in E-values is the highest in the PSM specifications at higher levels of correlation.

## Discussion

Controlling for covariates is essential to estimating treatment effects and PSMs are among the most common methodologies available to do so in observational research. Unfortunately, in almost all research studies there will be factors that are unobservable to the researcher. This limitation has motivated the development of numerous tools and best practices for designing, conducting, and assessing an observational analysis including multiple sensitivity analysis methods that can be applied to provide a measure of study robustness, including sub-cohort analyses, falsification tests, alternate specifications [8, 19–21]. However, the fact remains that balancing covariates in observational analyses does not guarantee balance in unobserved confounders which must be considered when applying sensitivity tests.

Our study examines the performance of a sensitivity analysis to estimate whether unobserved confounders would change the conclusion regarding the treatment effect estimate in the presence of amplification bias. We contribute to a body of literature that examines how methods to control for confounding may actually introduce bias [3, 22, 23]. We first confirm our data generating model results in increased imbalances in unobserved confounders after balancing observed covariates. We find that the treatment effect estimates relative to the true effect vary by specifics of the propensity score method (i.e., matching vs weighting and size of the matching caliper). We also document the effect of increasing strength of correlated unobservables on reducing the size of the final analytic sample. In practice, this has implications for the generalizability of the treatment effect estimate. We then extend our analysis to evaluate the impact of correlation among unobservables on the E-value calculation.

The appropriate application and interpretation of the E-value remains a point of contention in the literature. Besides the simple calculation and minimal assumptions, the E-value is intended to have a straightforward interpretation; a larger E-value indicates a treatment effect estimate is more robust to unmeasured confounding and a smaller E-value indicates more sensitivity to unmeasured confounding. In their review of the use of the E-value, Blum et al. (2020) highlight how E-value results are presented in publications with phrases such as “These results demonstrate that substantial unmeasured confounding would be needed to reduce the observed associations to null" [24]. However, there are no threshold values or formal guidance around appropriate conclusions that can be drawn based on the E-value. Critics of the E-value have suggested users should provide guidance to readers on the interpretation of the E-value in addition to a pre-specified value for an ‘explain away’ threshold [24]. It may be reasonable to place greater or lesser emphasis on the E-value depending on what is already known about unmeasured confounders and a research topic; however, no formal recommendations exist.

Although the purpose of the E-value is not to test for bias, our simulation study demonstrates that its interpretation can be affected by the presence and magnitude of amplification bias. Confirmation that a biased treatment effect estimate is not sensitive to unobserved confounding is not necessarily informative. It may confirm the existence of a causal effect but in many instances the magnitude of the effect also matters. We find that as the estimated treatment effect becomes more biased away from the true treatment effect, the E-value also increases. Thus, in this setting, the E-value incorrectly suggests that it is less likely that an unobserved confounder would change the conclusion of the analysis when in fact the treatment effect is biased toward finding an effect.

Our findings provide empirical support that an entire observational study protocol—both the main analyses and sensitivity analyses – must be informed by expertise on what is already known about potential unmeasured confounders in the context of a specific research question. Researchers who are aware of potential unobserved factors and possibly even a rough approximation of their magnitudes will be better able to determine the appropriate application of the E-value. This recommendation builds on a small but growing literature regarding E-values best practices. Blum and colleagues’ recommendations from their systematic literature review of E-values: users should provide guidance to readers on the interpretation of the E-value in addition to a pre-specified value for the ‘explain away’ threshold [24]. The recommendation is also consistent with VanderWeele and Mathur [25] who suggest that authors discuss potential unmeasured confounders and compare the E-value with covariate–outcome associations with prior literature. Our results demonstrate why this type of qualitative and quantitative bias assessment is needed.

Our paper has several limitations. First, our simulations used a single data generating process and only varied one aspect of the covariate correlation structure. It is impossible to know how much of an impact PSMs will have on the balance of unobserved covariates in any other data. In particular, our results do not generalize to data without variation of the unobserved covariates that affect treatment choice that is independent of the observed covariates. However, we expect that PSMs will always result in greater imbalance in unobserved covariates associated with only the treatment in settings where there is independent variation. Thus, the treatment effect estimate bias will be greater with PSMs relative to a regression approach in those instances but not for all data. In practice, researchers may consider multiple treatment effect estimation methods.

A second limitation is that our analysis of propensity score methods was limited to only two approaches. There have been numerous advances in PSMs and balancing methodologies more generally that may have different effects on the balance in unobservables [26–28]. The variation in effects and the inability to calculate exactly what the magnitude of the impact is for a study should also give researchers pause in applying a single measure to assess the robustness of their results.

## Conclusion

Bias in treatment effect estimates due to imbalance in unobserved confounders may result in the E-value suggesting a spurious confidence in results under various covariate adjustment methodologies.

## Data Availability

Datasets available from corresponding author upon reasonable request.

## Abbreviations

EHR: Electronic health record
GLM: Generalized linear model
IPTW: Inverse probability of treatment weighting
PSM: Propensity score methods
RCT: Randomized controlled trial
RWD: Real-world data
RWE: Real-world evidence
SMD: Standardized mean difference
